# 750. Launching the South Carolina Regional Infection Prevention Training (SCRIPT) Center: A Pilot in Interprofessional Education

**DOI:** 10.1093/ofid/ofad500.811

**Published:** 2023-11-27

**Authors:** Pamela Bailey, Sangita Dash, Shanetta Williams, Sabra Custer, Caroline Derrick, Katherine Green, Majdi N Al-Hasan, Julie Ann Justo

**Affiliations:** Prisma Health Richland - University of South Carolina, Columbia, South Carolina; University of South Carolina School of Medicine, Columbia, South Carolina; Prisma Health Midlands, Columbia, South Carolina; University of South Carolina School of Nursing, Columbia, South Carolina; University of South Carolina School of Medicine, Columbia, South Carolina; Prisma Health Midlands, Columbia, South Carolina; University of South Carolina School of Medicine, Columbia, South Carolina; Dartmouth Hitchcock Medical Center

## Abstract

**Background:**

Structured education of healthcare professionals around infection prevention practices and protocols is currently lacking. We launched a pilot program of an infection prevention interprofessional education (IPE) elective for students in their clinical years of medical, pharmacy, nursing, and physician assistant school.

**Methods:**

Both quantitative and qualitative assessments prior to initiation and at completion of the 2-week multidisciplinary interactive and practical IPE elective were performed. We assessed students’ confidence with specific infection prevention skills knowledge, and attitudes towards IPE, and obtained qualitative feedback. Wilcoxon signed rank test was used to compare assessment results before and after IPE elective.

**Results:**

Twelve students completed the IPE elective. There was a significant increase in Lickert scale rankings of students’ comfort level with various infection prevention concepts and skills after IPE (p< 0.001). Similarly, a significant improvement in students’ assessment of their IPE attitudes was noted after completion of the IPE elective (p=0.001).

Feedback comments included: “I really enjoyed learning the ‘why’ behind everything [because] it's made me comfortable passing the information to others and speaking up if needed--even still as a student”; “learned information I will use every day”; “people from different backgrounds”; “knowledgeable facilitators, excited to teach & facilitate learning.”

**Conclusion:**

Infection prevention education was impactful via the incorporation of an IPE elective into clinical rotations, and use of knowledgeable and adept teachers from multiple disciplines. Leaders emphasized the “why” behind protocols, and allowed students to practice the hands-on skills of infection prevention.

**Disclosures:**

**Julie Ann Justo, PharmD, MS, FIDSA, BCPS**, Gilead Sciences: Advisor/Consultant|Shionogi: Advisor/Consultant|Vaxart: Stocks/Bonds

